# Toward of Safer Phenylbutazone Derivatives by Exploration of Toxicity Mechanism

**DOI:** 10.3390/molecules24010143

**Published:** 2019-01-01

**Authors:** Rosivaldo S. Borges, Ivanete C. Palheta, Sirlene S. B. Ota, Roberto B. Morais, Valéria A. Barros, Ryan S. Ramos, Rai C. Silva, Josivan da S. Costa, Carlos H. T. P. Silva, Joaquín M. Campos, Cleydson B. R. Santos

**Affiliations:** 1 Núcleo de Estudos e Seleção de Moléculas Bioativas—NESBio, College of Pharmacy, Health Sciences Institute, Federal University of Pará, Belém 66075-110, PA, Brazil; ivapalheta@gmail.com (I.C.P.); sayuriota@gmail.com (S.S.B.O.); robertomorais16@hotmail.com.br (R.B.M.); valbarros43@yahoo.com.br (V.A.B.); 2 Programa de Pós-Graduação em Química Medicinal e Modelagem Molecular, Health Science Institute, Federal University of Pará, Belém 66075-110, PA, Brazil; camposchemistry@gmail.com; 3 Programa de Pós-Graduação em Biodiversidade e Biotecnologia—Rede BIONORTE, Federal University of Amapá, Macapá 68902-280, AP, Brazil; ryanquimico@hotmail.com; 4 Laboratorio de Modelagem e Química Computacional—LMQC, Federal University of Amapá, Department of Biological Sciences. Rod. Juscelino Kubitschek, Km 02, Macapá 68902-280, AP, Brazil; josivan.chemistry@gmail.com; 5 Laboratório Computacional de Química Farmacêutica, Faculdade de Ciências Farmacêuticas de Ribeirão Preto, University of Sao Paulo, São Paulo 14040-903, SP, Brazil; tomich@fcfrp.usp.br; 6 Department of Pharmaceutical Organic Chemistry, University of Granada, 18071 Granada, Spain; jmcampos@ugr.es

**Keywords:** phenylbutazone, DFT, electron transfer, metabolism, toxicity

## Abstract

A drug design for safer phenylbutazone was been explored by reactivity and docking studies involving single electron transfer mechanism, as well as toxicological predictions. Several approaches about its structural properties were performed through quantum chemistry calculations at the B3LYP level of theory, together with the 6-31+G(d,p) basis sets. Molecular orbital and ionization potential were associated to electron donation capacity. The spin densities contribution showed a preferential hydroxylation at the *para*-positions of phenyl ring when compared to other positions. In addition, on electron abstractions the aromatic hydroxylation has more impact than alkyl hydroxylation. Docking studies indicate that six structures **1**, **7**, **8** and **13**–**15** have potential for inhibiting human as well as murine COX-2, due to regions showing similar intermolecular interactions to the observed for the control compounds (indomethacin and refecoxib). Toxicity can be related to aromatic hydroxylation. In accordance to our calculations, the derivatives here proposed are potentially more active as well safer than phenylbutazone and only structures **8** and **13**–**15** were the most promising. Such results can explain the biological properties of phenylbutazone and support the design of potentially safer candidates.

## 1. Introduction

Phenylbutazone is a non-steroidal anti-inflammatory drug that has analgesic and antipyretic actions and can be used in some situations related do acute pain and muscular-skeletal disorders, such as in cases of ankylosing spondylitis and rheumatoid arthritis [1]. This medicine belongs to the group of pyrazolones, which are derived from enolic acid associated to its hydrogen α, α-dicabonylic [2].

It was synthesized in 1946 and introduced into clinical practice in 1949. However, in 1980 were attributed severe restrictions on the use of phenylbutazone in humans, because of the recognition that such a drug induced blood dyscrasias, which includes aplastic anemia, leukopenia, agranulocytosis and thrombocytopenia in some cases leading to death [3].

The toxicity of phenylbutazone is directly related to its mechanism of action, which shows non-selective effect for COX-1 isoform and may act on COX-2, thus the side effects are more evident in the organs and systems where COX-1 is found. The most known adverse reactions are diffused gastritis, gastric ulcers, venous thrombosis, nephritis and chronic renal injury [4,5]. As their toxicity depends on of metabolism and kinetics is a dose-dependent type, dosages up to two times above the recommended can result in fatal protein loss enteropathy, within 10 to 14 days [6]. Also, their main metabolites found were oxyphenbutazone and γ-hydroxy-phenylbutazone, where the first is active and second inactive. Only 1% of the administered dose is excreted in the urine as the parent drug while the majority (within 72%) is excreted in urine as metabolites and 10% is excreted in bile as well as metabolites [7].

Recently we have developed a theoretical prediction model of toxicity for pyrazolone drugs for agranulocytosis. Our study shows that this toxicity is mainly related to their ionization potential and nucleophilicity values, on electron transfer process [8]. Nevertheless, at moment the mechanism to phenylbutazone was not proposed. Thus, the study aimed to examine the molecular structure of phenylbutazone derivatives in a particular biological system, obtaining means for predicting the safer potential candidates.

## 2. Results and Discussion

### 2.1. Tautomerism Study

**Figure 1:**
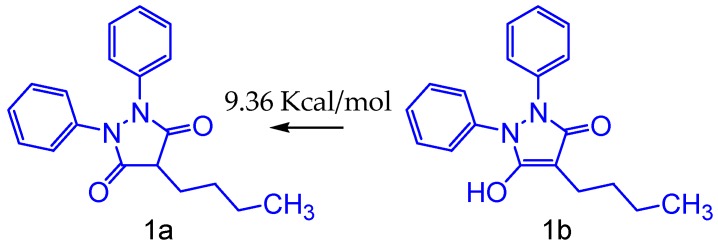
Tautomerism keto-enolic of phenylbutazone (**1a**) and phenylbutazone tautomers (**1b**).

The relative barrier is the energy difference of the most stable derivative to the less stable, that is, how much energy was needed to obtain the protonated form. In this study, we have investigated the phenylbutazone (**1a**) and phenylbutazone tautomers (**1b**) shown in Figure 1, depicting the keto-enol equilibrium for phenylbutazone. Results were obtained from quantum chemistry calculations at the theory level B3LYP and 6-31G+(d,p) basis set for the equilibrium structures, demonstrating that the conformation of the phenylbutazone molecule (**1a**) in the keto form is approximately 9.36 kcal/mol more stable than the other one. For the anionic form of phenylbutazone we observed greater planarity and rigidity of the five-membered ring (**1b**), on contrary to the proton form. In a qualitative analysis, we can infer a greater degree of electron delocalization, which could result in the appearance of a greater amount of resonance forms and consequent relocation of charges on the oxygen atoms.

Thus, the keto tautomer is more stable than the enol tautomer. Since phenylbutazone anion is reported as the active form in inflammation, it could act as free radical by a one-electron transfer mechanism, possibly via enol pathway [9]. However, despite this form to be unstable, any changes on pH medium should induce modification of the equilibrium relative to this tautomerism. The inflammation results in marked reductions of tissue pH by generation of acid derivatives as well as protons [10].

In addition, other pyrazole system showed the same behavior [11]. Hence for each calculation of phenylbutazone derivatives was realized from the keto tautomer. These conformations were used for electronic study of chemical reactivity and theoretical metabolism.

### 2.2. Molecular Simplification

**Figure 2:**
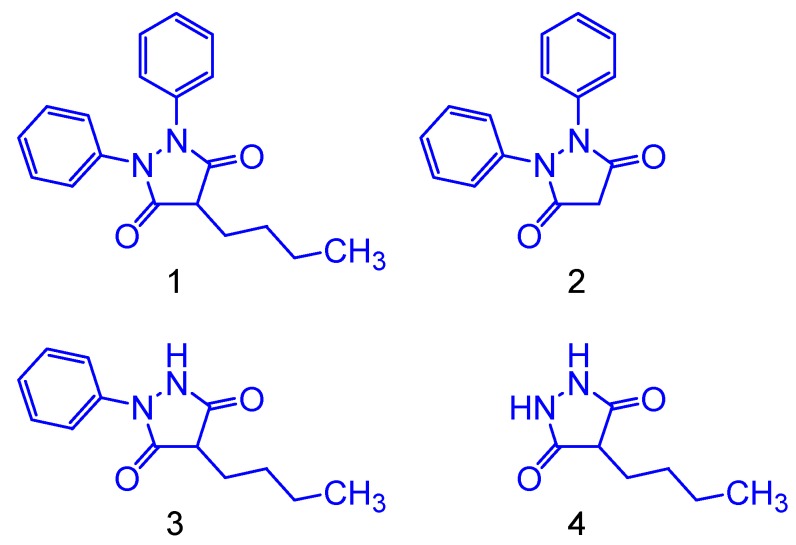
Molecular simplification of phenylbutazone.

**Figure 3:**
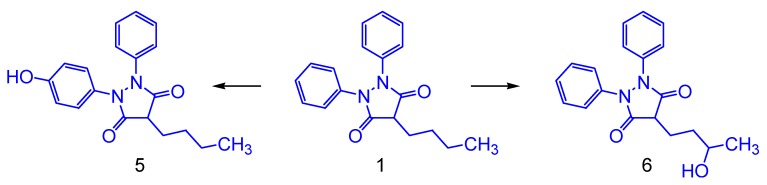
Molecular metabolism of phenylbutazone.

The electron donation capacity for the simplified phenylbutazone derivatives (Figure 2) were theoretically measured using HOMO, LUMO, Gap^L-H^ and ionization potential (IP) values. For the phenylbutazone compound, simplification of the phenyl and alkyl groups was carried out in order to investigate the structure-toxicity relationships at the pharmacophoric region: In compound **4**, the two phenyl rings were withdrawn due to the symmetry of the original molecule (phenylbutazone). The structures shown in Figure 3 are derived from oxidative metabolism, originating two main metabolites, through hydroxylation.

The HOMO and LUMO values are an important parameter of molecular structure related to their reactivity. They show all nucleophilic and electrophilic regions, respectively, around the molecule. The molecule which has the higher HOMO energy has strong donating electron ability. On the contrary, the higher LUMO value implies that the molecule is a good electron accepting compound [12]. The Gap^L-H^ values of these structures can indicate their chemical reactivity on electron transfers. The lowest Gap^L-H^ value is related to high chemical reactivity. Phenylbutazone showed HOMO value of −6.24 eV and Gap^L-H^ of 4.98 eV. Previous works have demonstrated a good tendency between HOMO and IP [13]. The IP value for the phenylbutazone is 177.46 kcal/mol. Additionally, the alkyl and phenyl moieties had been great influence on electron donation capacity for phenylbutazone derivatives. Indeed, our results showed that phenyl moiety has more impact than alkyl group due to HOMO decreases and by the increase of the Gap^L-H^ and IP values (Table 1).

Therefore, alkyl moieties and phenyl rings had a remarkable effect on electron donation capacity. In fact, the inflammation prostaglandin-mediated can to occur from an oxidation process by electron or hydrogen transfers followed for an oxygen addition. So, structures that have high electron donation capacity would have more inhibitory effect due to its antioxidant capacity [14].

### 2.3. Theoretical Metabolism

The oxidative metabolism of phenylbutazone (**1**) gives two main metabolites by means of hydroxylation, called oxyphenbutazone (**5**) and γ-hydroxyphenbutazone (**6**)—see Figure 3. The former is active and the second one is inactive [7]. Our calculations show that any hydroxylation can be related to changes on electronic behavior of phenylbutazone, such as electron donation capacity. However, the alkyl hydroxylation at *N*-buthyl position can be related to changes on solubility due to the loss of lipophilic interaction at the pocket of enzyme responsible for arachidonic acid oxidation.

In fact, oxyphenbutazone (**5**) has demonstrated more electron donation capacity than phenylbutazone. As we can see the best values for HOMO of −6.04 eV, Gap^L-H^ of 4.83 eV and IP of 166.43 kcal/mol for **5**. These values would be related to its biological activity [15]. Likewise, these properties change for hydroxylated alkyl derivative **6** is few when compared to oxyphenbutazone (**5**). In addition, the high electron donation capacity of oxyphenbutazone can be related to their toxicity increases [15]. The aromatic rings are the best nucleophilic moieties especially due to nitrogen atoms. As a result, after an electron transfer the most reactive positions can be localized by spin densities calculations. It may be used to prediction a regioselective hydroxylation.

**Figure 4:**
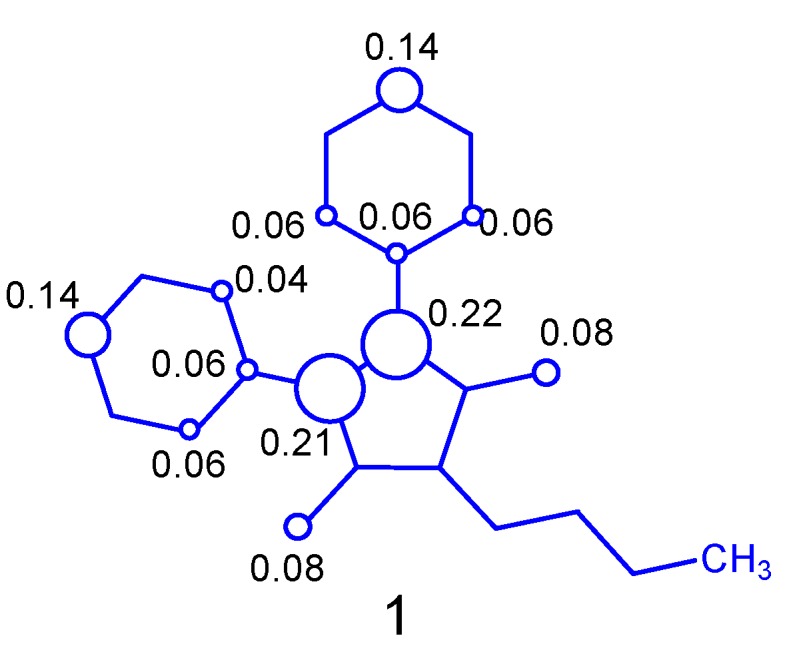
Spin densities contribution of phenylbutazone cation free radical.

Therefore, the calculated spin density to initial electron abstraction on phenylbutazone (Figure 4) shows the main contributions from the nitrogen atoms (0.21 and 0.22), carbon atoms at the *para*-positions (0.14) and oxygen atom at the carbonyl groups (0.08). The contribution from the other atoms is almost an order of magnitude minor. These results showed that the reactivity increase at the *para*-positions (0.14) and explain the preferential regioselective radical attack of hydroxyl group to form aromatic hydroxylated-type derivatives such as oxyphenbutazone.

### 2.4. Proposed Phenylbutazone Derivatives

Among the theoretical properties of phenylbutazone, the most remarkable are their high nucleophility, electron donation capacity, lipophilicity and molecular volume. These values can be observed through electronic, solubility and structural parameters in Table 2.

**Figure 5:**
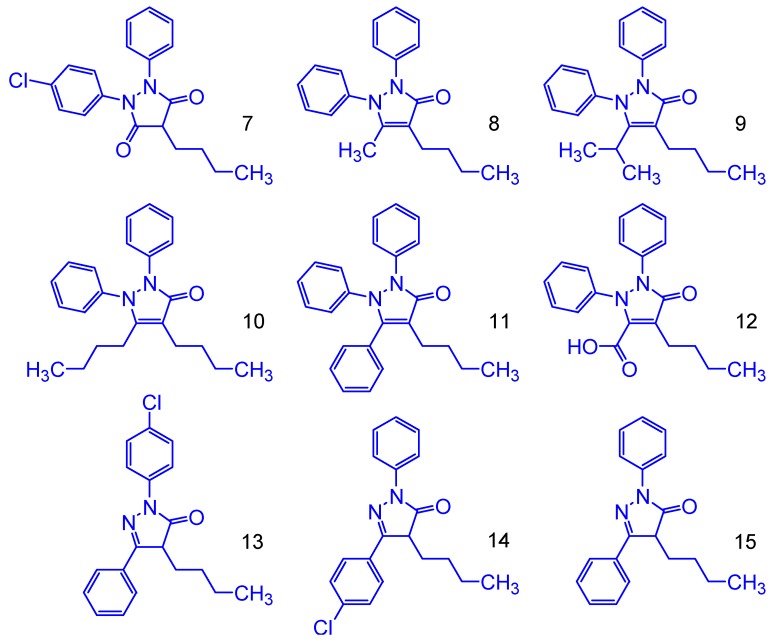
Molecular structure of proposed phenylbutazone derivatives.

Phenylbutazone showed HOMO value of −6.24 eV and Gap^L-H^ of 4.98 eV. It is IP, Log P and Vol values are 177.46 kcal/mol, 4.22 and 941.89 Å^3^, respectively. Based on them, nine derivatives were proposed by the combination among phenylbutazone, edaravone [16] and antipirine (Figure 5). The chloro moiety was added as metabolism inhibitor at *para*-position at the phenyl ring hydroxylation reactions [17].

Results obtained indicate that all proposed structures have more electron donation capacity than phenylbutazone, in accordance to the HOMO, Gap^L-H^, IP and ΔEiso values. Likewise, all structures have similar lipophilicity and high molecular volume, when compared to phenylbutazone. Unfortunately, despite of all the proposed structures to be like actives and safer, in potential, other properties need to be considered, such as bioavailability and other pharmacokinetic parameters. However, validations will be completed only after synthesis and the respective biological evaluations. In addition, all the theoretical values obtained for our derivatives are very close of the observed for phenylbutazone and they do not violate the Lipinski RO5 rule [18].

### 2.5. Molecular Docking Study

For docking method validation, the indomethacin and rofecoxib structures with crystallographic information were submitted to docking until the folding found by software that was similar to the crystallographic information.

**Figure 6:**
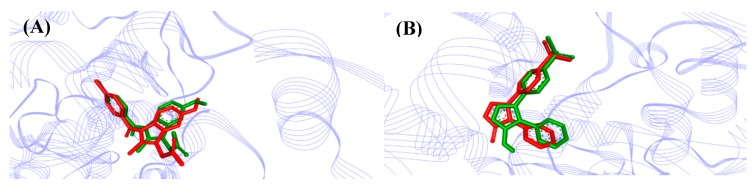
AutoDock validation of (**A**) COX-2 (*Mus musculus*, PDB 4COX) and (**B**) COX-2 (*Homo sapiens*, PDB 5KIR) with respective crystallographic ligands.

The comparison between crystallographic ligands IMN and RCX (red color) and best-predicted docking conformation (green), can be seen in Figure 6 that show a great pose (orientation + conformation) where the RMSD values were 0.75 and 1.04 Å for indomethacin and rofecoxib, respectively.

According to Gowthaman et al. (2008) [19] and Hevener et al. (2009) [20], the binding mode prediction using the docking, affirm that when the RMSD is less than 2.0 Å on the crystallographic pose of ligand is considered successful. Therefore, our results with the methodological proposal using these parameters are optimal and satisfactory.

Using the docking methodology here selected, we have identified a potential binding mode for the compound able to interact with the active site of COX-2, similar to the crystallographic pose observed for indomethacin (PDB ID 4COX), that is, around the α-helix located between the amino acid residues 118–121, 348–352 and 522–526, as well as around the β-sheet located between the amino acid residues 353–354 and 527. For the ligand, it is possible to see common hydrogen bonds formed with Tyr355 and Arg120 residues. There is also a hydrophobic interaction with Val349, Ser353, Leu352, Val523 and Ala527 residues, according to studies found in the literature [21].

Also, the interaction sites described for rofecoxib ((PDB ID 5KIR) are located around the α-helix between the amino acid residues 88–91, as well as the amino acids residues located between 352–354 and 515–519 around the β-sheet, according to studies carried out by Orlando et al. (2016) [22]. The validation was accepted despite of the minor deviation observed between the poses, because the two crystallographic poses are possible.

In order to evaluate whether the changes made would lead to a higher binding affinity than the inhibitor respective to each targeted COX-2 structure, that is, from the *Mus musculus* and *Homo sapiens*, we docked each COX-2 structure, its specific ligand with phenylbutazone (structure **1**) and phenylbutazone derivatives (structures **7**–**15**) in Auto-Dock/Vina software. 

We observed that indomethacin (IMN) as ligand showed higher binding affinity (−9.5 kcal/mol) to COX-2 (organism *Mus musculus*) than phenylbutazone (structure **1**) and phenylbutazone derivatives (structures **7**–**15**). However, when comparing the structures donated in *Mus musculus* organism, rofecoxib had a lower affinity value (−7.8 kcal/mol) when compared to phenylbutazone and their derivatives.

**Figure 7:**
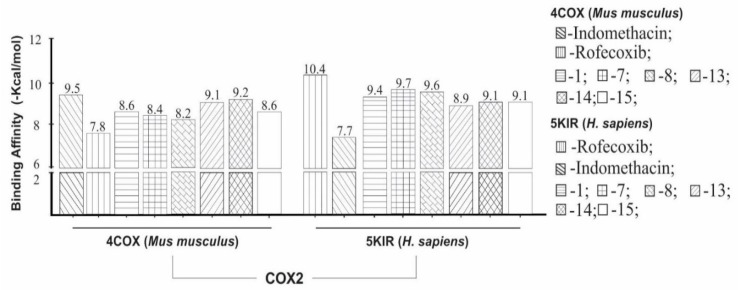
Binding affinity provided by AutoDock/Vina software of the phenylbutazone derivatives (structures **1**, **7**–**15**) and standard ligands. Ligand control for COX-2 organism *Mus musculus* and *Homo sapiens* were indomethacin and rofecoxib.

However, the structure indicates a theoretical higher binding affinity of −9.2 kcal/mol, when compared to the here investigated structures and it had a variation of ±0.3 kcal/mol in comparison to indomethacin (IMN), whereas the other ones had a variation ranging from ±1.3 to ±0.4 kcal/mol. All these values are shown in Figure 7.

Furthermore, we observed that ligand RCX showed a higher binding affinity values (−10.4 kcal/mol) to COX-2 (*Homo sapiens*), when compared to phenylbutazone (**1**) and phenylbutazone derivatives (structures **7**–**15**) as well. Indomethacin showed the lowest affinity (−7.7 kcal/mol). However, compound **7** had a higher binding affinity (−9.7 kcal/mol) regarding to the studied structures and had a variation ±0.7 kcal/mol in comparison to RCX and the other one structures had a variation ranging from ±1.5 to ±0.8 kcal/mol.

With these data, we propose that the phenylbutazone (**1**) and phenylbutazone derivatives (**7**–**15**) are able to make binding interaction on both organisms (*Mus musculus* and *Homo sapiens*). However, **1**, **13**–**15** derivatives have greater binding affinity to enzyme from *Mus musculus* but **1**, **7**, **8**, **14** and **15** derivatives showed greater binding affinity to the human enzyme.

According to the studies of Beretta et al. (2005) [23], the examination of IC_80_ values of the four non-steroidal anti-inflammatory drugs among them phenylbutazone (+134.4%) and flunixin (+29.7%). Phenylbutazone show greater selectivity for COX-2 in accordance to 50% inhibitory concentration. In fact, we suggested that phenylbutazone derivatives has both anti-inflammatory and peripheral analgesic effects and Beretta et al. (2005) [23] has demonstrated that phenylbutazone and flunixin in horses when compared to meloxicam, retains a best selectivity at higher concentrations. We can also to observe higher interaction numbers of indomethacin which is a potent non-selective inhibitor of the enzyme cyclooxygenase (COX), being a fundamental element of the cascade of arachidonic acid, the metabolic pathway that allows the synthesis of prostaglandins and thromboxanes.

**Figure 8:**
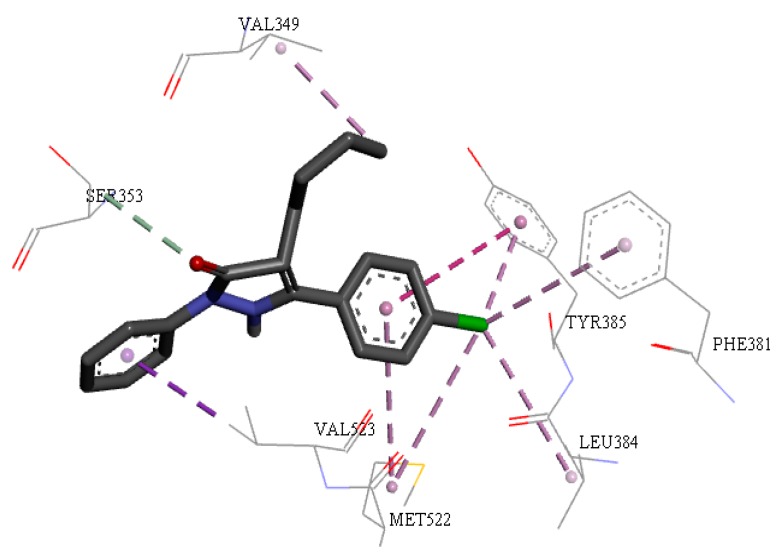
Interactions of structure between **14** and COX-2 of *Mus musculus*. Hydrophobic effect of alkyl or Pi-alkyl, Pi-sulfur, hydrogen bonds and Pi-Pi interactions are shown.

Figure 8 shows all interactions for individually docked structure **14** that had similar interactions to the active sites of COX-2 (*Mus musculus*) for indomethacin to around the alpha helix between the amino acids residues Ser353, Val523 and Gly526, in β-leaf the amino acids residue Ala527. There is also a hydrophobic interaction with residues Val349, Ser353, Tyr385, Val523, Met522 and Ala527.

**Figure 9:**
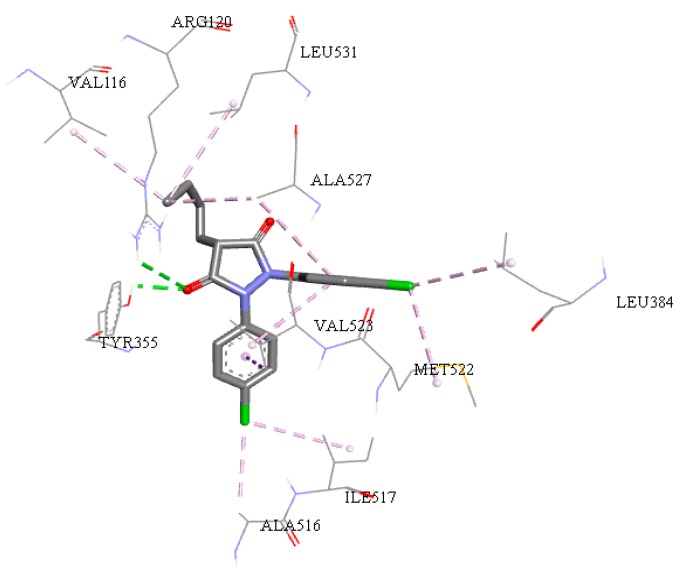
Interactions of structure **7** (COX-2 organism *Homo sapiens*). Hydrophobic interactions alkyl, Pi-alky, Pi-sulfur, hydrogen bonds and Pi-Pi interactions can be seen.

All amino-acids residues between ligands with therapeutic targets COX-2 for the *Mus musculus* organism are shown in Tables S1 and S2 (see supplementary material). Similarly, analyzing the interaction sites for structure **7**, as shown in the Figure 9 and comparing with the interaction sites rofecoxib, we observed similar results between *Homo sapiens* and *Mus musculus* organism. Both active sites of COX-2 have some amino-acid residues around the α-helix identified by the residue Arg120 and Val116, for β-leaf the amino acids residues Tyr 355 and Leu384, Ala 516 and Met522. In addition, the residues Val523 and Ala527 were identified close to the active site.

Quantitative data of distances and binding free energies (ΔG) between the phenylbutazone derivatives and COX-2 for both receptors of *Mus musculus* and *Homo sapiens* organisms can be seen in Tables S1–S4 (see supplementary material). It is possible to verify that, among the template structures, such as indomethacin (IMN) and rofecoxib (RCX) inhibitors, the increase in the number of interactions resulted in diminution of the binding free energy, which indicates a higher degree of spontaneity of the interactions. This type of analysis provides additional information of features of the compounds to get a better understanding of the chemical behavior for new drugs.

This effect is noticeable for phenylbutazone derivatives with binding free energy similar to the observed for the template structures. In fact, common interactions with the amino-acids occurred in all the analyzed structures and they can indicate that they play a relevant play in the anti-inflammatory activity for the structures here investigated—Tables S1–S4 (see supplementary material).

Comparing phenylbutazone derivatives with the template molecules indomethacin and rofecoxib, we can observe that diminution of the binding free energy is similar because they have two different interactions, one with Leu352 and another with Ser353. Our results show that the interactions are a favorable factor for lowest values of ΔG here obtained.

The alkyl or aryl interactions have strong hydrophobic characteristic and they are related to favorable entropic factors can promote the lowering of the free energy of ligand-receptor binding and all the tested ligands were capable of forming hydrogen interactions with residues at their respective binding sites [24,25,26,27,28]. This result confirms that proposed phenylbutazone derivatives have in silico anti-inflammatory activity via COX inhibition, as well as, in silico study is a valuable tool as a support for in vivo assays and from the in silico analysis, mechanisms can be clarified at the molecular level.

In the rational drug design process, availability of 3D (three-dimensional) protein structures is crucially important. The X-ray crystallography, considered an efficient tool in determining protein 3D poses, is a time-consuming and expensive method. In addition, certain proteins cannot be successfully crystallized or are difficult to crystallize, for example the membrane proteins. Therefore, a very low number of membrane proteins structures has been determined. The recent breakthroughs indicate that NMR is indeed a very powerful tool in determining the 3D structures of membrane proteins [29,30,31,32] but it is also time-consuming and costly. To acquire the structural information in a timely manner, a series of 3D protein structures were developed by means of homology (or comparative) technique and were found very useful for drug development [33,34,35].

### 2.6. Toxicological Properties

In this present study, only structures **1**, **7**, **8**, **14** and **15** showed good results of molecular docking as well as predictions of toxicological properties, in comparison to the results obtained for the template molecules (Indomethacin, Refocoxib and Phenylbutazone). The toxicological properties of the structures containing toxicity alarms are shown in Table 3. It is noted that indomethacin in the toxicity analysis did not show any alert, a fact that could be due to the low concentration in which the compound acts inside the active site. Analysis of the toxicological properties allowed us to observe that all the structures point out toxicity alerts characterized as “plausible” or “acceptable”—see Table 3.

Refecoxib showed an alert of hepatotoxicity respective to furan derivatives. Many of these compounds have been reported to cause a dose-related hepatic necrosis in rodents including furosemide to produce midzonal to centrilobular necrosis in mice and rats [36,37,38,39]. The mechanism of toxicity is proposed to involve oxidative ring opening of the furan ring leading to formation of reactive intermediates, which can alkylate proteins or form adducts with DNA [40]. Evidence of liver toxicity for furan-containing compounds in humans is limited. For example furosemide has not been associated with liver effects in humans but it has caused jaundice and attention has been drawn to its potential hepatotoxicity in case of high dose administration to patients with renal failure [38,41,42]. Furan is a liver toxicant and hepatocarcinogen in rodents [43,44].

The hepatotoxicity of furan and related compounds is thought to result from the formation of chemically reactive metabolites [37,40]. The oxidative opening of the furan ring is primarily catalyzed by cytochrome P450s and is thought to proceed via the initial formation of a furan-epoxide. The scission of the ring leads to the formation of an electrophilic alpha, β-unsaturated dicarbonyl moiety, which can covalently bind to macromolecules via a Michael-type addition [40]. 

The scope of this alert has been defined by the common structural features of the active compounds in this class, namely the furan ring moiety. The monosubstituted 2- and 3-methylfurans are excluded on the basis they can be readily oxidized to carboxylic acids and excreted without causing toxic effects as supported by experimental studies in rodents [38,39]. Based on this data, carboxylic acids and their derivatives are also excluded from the furan ring. Nitro and alkyl amine compounds are also omitted as they are thought to induce toxicity via a different mechanism [45].

In Table 3, structures **1** and **7** are shown containing alerts of hepatotoxicity for 1,2-diphenyl-3,5-pyrazolidinedione derivatives. During their use as non-steroidal anti-inflammatory drugs (NSAIDs), this class has been reported to cause necrosis, steatosis and granulomas in the liver [26]. The mechanism of hepatotoxicity is thought to be an immune-mediated reaction but a dose related intrinsic effect has also been reported for phenylbutazone [46].

Phenylbutazone and its pharmacologically active metabolites oxyphenbutazone and kebuzone are used as NSAIDs. They may cause various side effects including several forms of hepatic injury occurring during treatment. In many reports of human liver toxicity by phenylbutazone (daily doses varying from 300 mg to 800 mg) the hepatocellular injury was described as being acute and the severity ranged from mild to moderate with or without cholestasis [36,46].

Phenylbutazone has been reported to cause steatosis, cholestasis and centrilobular necrosis, often with ballooning degeneration and hypertrophy of Kupffer cells. Noncaseating granulomas in the portal and periportal areas are observed in cases of hypersensitivity to this drug [46]. In addition, it has been reported that its active metabolites cause similar toxic effects [47,48]. Idiosyncratic liver injury was reported after administration of feprazone, (daily dose of 600 mg) when biopsies of individuals showed inflammation of the portal tract and parenchyma [49]. Sulfinpyrazone, an uricosuric agent, has been described as an inducer of microsomal enzymes and shown to cause hepatic injury in isolated cases [50].

Mice exposed to phenylbutazone by gavage (150–300 mg/kg) during a 2 years study were found to have hepatomegaly, steatosis, cellular degeneration and coagulative necrosis [51]. The mechanism of hepatotoxicity is not clear for this class but is reported to be mostly idiosyncratic, although the toxicity of phenylbutazone has been reported to have two components. The intrinsic toxicity of phenylbutazone is thought to cause cholestasis and necrosis, while cases of hypersensitivity indicated by the presence of granulomas, are thought to be due to idiosyncratic reactions [46].

Results obtained for compounds **1**, **7**, **8**, **13**–**15** indicating toxicity alerts also include coverage for hydrazone precursors of hydrazines. The presence of a skin sensitization structural alert within a molecule indicates the molecule has the potential to cause skin sensitization. Whether or not the molecule will be a skin sensitizer will also depend upon its percutaneous absorption. Generally, small lipophilic molecules are more readily absorbed into the skin and are therefore more likely to cause sensitization [52,53,54]—see Table 3.

## 3. Materials and Methods

### 3.1. Theoretical

Initially, the calculations of Density Functional Theory (DFT) have also been employed in the determination of geometry and physic-chemical properties such as partition oil-water (Log P) and volume (V) for the phenylbutazone and its related derivatives [55]. It was done using computational program Gaussian 09 molecular package [56]. In order to understand the interaction way at the molecular level on phenylbutazone derivatives, their structural and electronic properties were obtained by parameters of reactivity and theoretical properties such as energies occupied molecular orbital of highest energy (HOMO) and the energy unoccupied molecular orbital of lowest energy molecular orbital (LUMO) and ionization potential (IP).

To examine the reactivity of the calculated compounds (HOMO-LUMO) Gap^L-H^ obtained by the difference in energy between the HOMO and LUMO [57] (Equation (1)).

Gap^L-H^ = *E*_LUMO_ − *E*_HOMO_ (1)

The DFT method was employed, using the B3LYP functional hybrid [58,59] and the 6-31+G(d,p) basis set to phenylbutazone derivatives (PD) which act as electron donating group. The ionization potential (IP) was calculated as the energy difference between the neutral molecule (*E*PD) and the free radical cation (*E*PD^•+^) (Equation (2)). In addition, the stabilization energy (ΔEiso) was calculated by means of the energy’s difference between the phenylbutazone cation free radical (*E*PBT^•+^) plus neutral form of derivatives (*E*PD) and the respective phenylbutazone neutral molecule (*E*PBT) plus cation free radical of their derivatives (*E*PD^•+^) (Equation (3)).

IP = *E*PD^•+^ − *E*PD (2)

ΔEiso = (*E*PBT^•+^ + *E*PD) − (*E*PBT + *E*PD^•+^) (3)

### 3.2. Molecular Docking Simulations Study

The cyclooxygenase-2 (COX-2) structures resolved by X-ray diffraction was downloaded from the Protein Data Bank (PDB) with PDB codes 4COX (*Mus musculus*) and 5KIR (*Homo sapiens*) with resolution of 2.9 and 2.7 Å, respectively.

The control standard ligands used in docking molecular study were indomethacin (IMN) and rofecoxib (RCX) downloaded at PDB server in sdf format ensuring the bioactive conformation. All phenylbutazone derivatives structures were optimized using the density functional theory method (DFT) in theory level B3LYP/6-31+G(d,p). Also, the frequencies were also calculated using B3LYP/6-31+G(d,p) and observed that there were no negative frequencies, thereby ensuring that local minimum energy structure [60]. The phenylbutazone derivatives and therapeutic targets structures for molecular docking study were prepared using the software Discovery Studio 5.0 (San Diego, CA, USA) [61], removing the water molecules and for docking validations were downloaded with the therapeutic targets crystal structures and crystallographic inhibitors were used in molecular docking study, as well as, in validation through of the root-mean-square deviation (RMSD) value between the experimental data with computational data, according to studies carried out by Macêdo et al. (2015) [62] and Federico et al. (2017) [63].

The indomethacin structure is a non-selective inhibitor (*Mus musculus*, PDB 4COX) and rofecoxib structure bound to human COX-2 (*Homo sapiens*, PDB 5KIR) were used via AutoDock 4.2/Vina 1.1.2 (Scripps Research Ins., San Diego, CA, USA) by graphical interface Pyrx (version 0.8.30) with default parameters via the genetic algorithm, following the protocol described previously by Padilha et al. (2016), Pereira et al. (2018) and Costa et al. (2018) [64,65,66]. The population size was 100, selection-pressure was 1.1, the number of operations was 10,000, the number of islands was 1, the niche size was 2, operator weights for migrate was 0, mutate was 100 and crossover was 100. Spatial coordinates used for the center of the grid is described in Table 4.

The coordinates of the grid box located at the pocket of interest sere selected based on interactions observed between inhibitor and enzyme, inside a 10 Å radius sphere then defined. Ten solutions were calculated for each phenylbutazone derivatives and minimum binding energy conformations were analyzed.

Docking studies both COX-2 and their complexed specific ligands were carried out using the AutoDock 4.2/Vina 1.1.2 and PyRx 0.8.30 software. Spatial coordinates for PDB ID 4COX and PDB ID 5KIR were chosen according to the interaction observed between the two COX enzymes and their respective original ligands. The visualizations and distance of ligands interactions with COX were in Discovery Studio 5.0.

The energy score function was used to assess the binding free energy (ΔG) of interactions between COX and phenylbutazone derivatives in PyRx 0.8.30. Analysis of each pose (conformation + orientation) of the ligands were also taken into account for selection of the best ΔG values as well as binding affinity, using AutoDock 4.2/Vina 1.1.2, in order to evaluate the selectivity (and binding affinity) of the phenylbutazone derivatives regarding the targeted COX-2 structures here used (from *Mus musculus* and *Homo sapiens*). Both the indomethacin and rofecoxib were used as templates or positive control controls for inhibiting COX-2 because they are known potent COX-2 inhibitors.

### 3.3. Toxicological Predictions

Toxicity profile of the structures was evaluated using the Deductive Estimation of Risk from Existing Knowledge (DEREK) 10.0.2 software (Leeds, UK) [67]. We have considered DEREK alerts of toxicity involving the human, mouse and rat species. DEREK makes the prediction of toxicity of the structures in a qualitative way, since it is an expert system that focuses attention on the toxic action of chemical compounds. The system performs this analysis based on implemented rules and depicts the relationship between a structural feature and a toxicophoric group present in the compounds as possible inducers of certain types of toxicity. It is considered that, in addition to toxicity, DEREK can identify specific aspects related to carcinogenicity, mutagenicity, skin sensitization, irritation, teratogenicity and neurotoxicity [68,69].

## 4. Conclusions

Our results using DFT/B3LYP/6-31+G(d,p) calculations showed that the molecular orbitals, ionization potential and spin density contributions of phenylbutazone can be used to explain their oxidation prediction and chemical reactivity. Both phenyl rings have more influence under electron donation capacity than alkyl moiety. Aromatic hydroxylation ring has more impact on electron donation capacity than alkyl hydroxylation. The spin densities contribution showed that a regioselective hydroxylation is more favored at the *para*-positions of phenyl ring than other positions. Docking results here obtained indicate that compounds **1**, **7**, **8** and **13**–**15** have potential capacity of COX-2 inhibition in human and mice enzyme as well, due to contain similar interactions to the observed for the templates or control compounds (indomethacin and refecoxib). The toxicity could be here related to an aromatic hydroxylation. In accordance to our calculations, the proposed derivatives are potentially more active and safer than phenylbutazone and only compounds **8** and **13**–**15** were the most promising. These results could explain the biological properties observed for phenylbutazone and they support the design of novel and safer derivative candidates.

## Figures and Tables

**Table 1:** Theoretical properties of phenylbutazone derivatives.

Derivatives	HOMO (eV)	LUMO (eV)	GAP^L-H^ (eV)	IP (kcal/mol)
**1**	−6.24	−1.25	4.98	177.46
**2**	−6.31	−1.31	5.01	180.32
**3**	−6.35	−1.30	5.05	185.11
**4**	−7.15	−1.21	5.94	215.45
**5**	−6.04	−1.20	4.83	166.43
**6**	−6.20	−1.22	4.99	170.12

**Table 2:** Theoretical properties of proposed phenylbutazone derivatives.

Derivatives	HOMO (eV)	LUMO (eV)	GAP^L-H^ (eV)	IP (kcal/mol)	ΔEiso (kcal/mol)	Log P	Vol (Å^3^)
**1**	−6.24	−1.25	4.98	177.46	0	4.22	941.89
**7**	−6.44	−1.55	4.88	174.44	−3.01	5.26	1027.15
**8**	−5.83	−0.94	4.89	161.07	−16.38	3.94	979.66
**9**	−5.83	−0.99	4.83	159.79	−17.66	4.81	1051.55
**10**	−5.80	−0.97	4.83	159.54	−17.91	5.20	1119.93
**11**	−5.91	−1.46	4.44	159.69	−17.76	5.37	1030.56
**12**	−6.11	−2.21	3.90	168.09	−9.37	3.39	1010.77
**13**	−6.02	−2.10	3.91	167.21	−10.25	5.37	960.47
**14**	−6.02	−2.02	4.00	167.15	−10.30	5.37	963.55
**15**	−5.93	−1.90	4.02	166.03	−11.42	4.85	920.66

**Table 3:** Predictions of the toxicological properties of structures investigated.

Structures	Toxicity Prediction Alert (Lhasa Prediction)	Toxicophoric Group	Toxicity Alert	Toxicity Prediction (Custom Prediction)
Indometacin	-	-	-	Nothing to declare
Refecoxib	Hepatotoxicity in human, mouse and rat	Furan	PLAUSIBLE	Nothing to declare
Phenylbutazone (**1**)	Hepatotoxicity in human and mouse	1,2-Diphenyl-3,5-pyrazolidinedione derivative	CERTAIN	Nothing to declare
	Hepatotoxicity in rat	1,2-Diphenyl-3,5-pyrazolidinedione derivative	PROBABLE	
	Skin sensitization in human, mouse and rat	Hydrazine or precursor	PLAUSIBLE	
**7**	Hepatotoxicity in human, mouse and rat	1,2-Diphenyl-3,5-pyrazolidinedione derivative	PLAUSIBLE	Nothing to declare
	Skin sensitization in human, mouse and rat	Hydrazine or precursor	PLAUSIBLE	
**8**	Skin sensitization in human, mouse and rat	Hydrazine or precursor	PLAUSIBLE	Nothing to declare
**13**	Skin sensitization in human, mouse and rat	Hydrazine or precursor	PLAUSIBLE	Nothing to declare
**14**	Skin sensitization in human, mouse and rat	Hydrazine or precursor	PLAUSIBLE	Nothing to declare
**15**	Skin sensitization in human, mouse and rat	Hydrazine or precursor	PLAUSIBLE	Nothing to declare

**Table 4:** Data from protocols used for molecular docking validation purposes.

Receptor	Ligand	Ligand Coordinates of the Grid Center	Grid Size (Points)
COX-2 (PDB code: 4COX) *Mus musculus*	Indomethacin	X = 11.5685 Y = 12.3903 Z = 7.2430	26 x 30 y 28 z
COX-2 (PDB code: 5KIR) *Homo sapiens*	Rofecoxib	X = 26.6293 Y = 40.1834 Z = 3.8484	26 x 30 y 28 z

## Supplementary Materials

The following are available online.

## References

[1] Lees P., Toutain P. (2013). Pharmacokinetics, pharmacodynamics, metabolism, toxicology and residues of phenylbutazone in humans and horses. Vet. J..

[2] MacAllister C.G., Morgan S.J., Borne A.T., Pollet R.A.J. (1993). Comparison of adverse effects of phenylbutazone, flunixin meglumine. and ketoprofen in horses. J. Am. Vet. Med. Assoc..

[3] Lees P., Higgins A.J. (1985). Clinical pharmacology and therapeutic uses of non-steroidal anti-inflammatory drugs in the horse. Equine Vet. J..

[4] Mathews K.A. (2002). Non-steroidal anti-inflammatory analgesics: A review of current practice. J. Vet. Emerg. Cris. Care.

[5] Fitzpatrick J.L., Nolan A.M., Lees P., May S.A., Andrews A.H., Blowey R.W., Boyd H., Eddy R.G. (2004). Inflammation and pain. Bovine Medicine Diseases Husandry Cattle.

[6] Collins L.G., Tyler D.E. (1984). Phenylbutazone toxicosis in the horse: A clinical study. J. Am. Vet. Med. Assoc..

[7] Triggs E.J., Whyatt P.L., Eckert G. (1977). Bioavailability evaluation of an enteric-coated phenylbutazone formulation. and cross-over comparison with a sugar-coated product. Med. J. Aust..

[8] Silva B.H.S., Barros T.G., Barros C.A.L., Vieira J.L.F., Borges R.S. (2011). An electronic study of biphenyl derivatives chlorinated. J. Comp. Theor. Nanosc..

[9] Levy G.N. (1997). Prostaglandin H synthases. nonsteroidal anti-inflammatory drugs and colon cancer. FASEB J..

[10] Kidd B.L., Urban L.A. (2001). Mechanisms of inflammatory pain. Br. J. Anaesth..

[11] Queiroz A.N., Mendes A.P.S., Leal M.R.S., Chaves Neto A.M.J., Borges R.S. (2010). Tautomerism and radical-scavenging activity of edaravone by DFT methods. J. Comp. Threoret. Nanosc..

[12] Antonczak S. (2008). Electronic description of four flavonoids revisited by DFT method. J. Mol. Struct. TheoChem..

[13] Queiroz A.N., Gomes B.A.Q., Moraes W.M.J., Borges R.S. (2009). A theoretical antioxidant pharmacophore for resveratrol. Eur. J. Med. Chem..

[14] Lobo V., Patil A., Phatak A., Chandra N. (2010). Free radicals antioxidants and functional foods: Impact on human health. Pharmacogn. Rev..

[15] Wood C.M., Schlenk D., Benson W.H. (2001). Toxic responses of the gill. Target Organ Toxicity in Marine and Freshwater Teleosts.

[16] Borges R.S., Queiroz A.N., Mendes A.P.S., Araújo S.C., França L.C.S., Franco E.C.S., Gomes-Leal W., Silva A.B.F. (2012). Density functional theory (DFT) study of edaravone derivatives as antioxidants. Int. J. Mol. Sci..

[17] Carvalho E.S., Santa-Brígida S.A., Queiroz A.N., Silva J.R., Silva O.P.P., Barros C.A.L., Borges R.S. (2017). Edaravone toxicity can be related to redox properties of their oxidized derivatives. Chem. Data Collect..

[18] Lipinski C.A., Lombardo F., Dominy B.W., Feeney P.J. (2001). Experimental and computational approaches to estimate solubility and permeability in drug discovery and development settings. Adv. Drug Deliv. Rev..

[19] Gowtham U., Jayakanthan M., Sundar D. (2008). Molecular docking studies of dithionitrobenzoic acid and its related compounds to protein disulfide isomerase: Computational screening of inhibitors to HIV-1 entry. BMC Bioinform..

[20] Hevener K.E., Zhao W., Ball D.M., Babaoglu K., Qi J., White S.W., Lee R.E. (2009). Validation of molecular docking programs for virtual screening against dihydropteroate synthase. J. Chem. Inform. Model..

[21] Kurumbail R.G., Stevens A.M., Gierse J.K., Mcdonald J.J., Stegeman R.A., Pak J.Y., Gildehaus D., Iyashiro J.M., Penning T.D., Seibert K. (1996). Structural basis for selective inhibition of cyclooxygenase-2 by anti-inflammatory agents. Nature.

[22] Orlando B.J., Malkowski M.G. (2016). Crystal structure of rofecoxib bound to human cyclooxygenase-2. Acta Crystallogr..

[23] Beretta C., Garavaglia G., Cavalli M. (2005). COX-1 and COX-2 inhibition in horse blood by phenylbutazone. flunixin. carprofen and meloxicam: An in vitro analysis. Pharm. Res..

[24] Costa J.S., Costa K.S.L., Cruzb J.V., Ramos R.S., Silva L.B., Brasil D.S.B., Tomich C.S., Rodrigues C.B., Macêdo W.J.C. (2018). Virtual screening and statistical analysis in the design of new caffeine analogues molecules with potential epithelial anticancer activity. Curr. Pharm. Des..

[25] Cruz J.V., Neto M.F.A., Silva L.B., Ramos R., Costa J., Brasil D.S.B., Lobato C.C., Costa G.V., Bittencourt J.A.H.M., Silva C.H.T.P. (2018). Identification of novel protein kinase receptor type 2 inhibitors using pharmacophore and structure-based virtual screening. Molecules.

[26] Santos C.B.R., Ramos R.S., Ortiza B.L.S., Silva G.M., Giuliatti S., Navarrete J.L.A., Carvalho J.C.T. (2018). Oil from the fruits of *Pterodon emarginatus* Vog.: A traditional anti-inflammatory. Study combining in vivo and in silico. J. Ethnopharmacol..

[27] Teles Fujishima M.A., Silva N.S.R., Ramos R.S., Batista Ferreira E.F., Santos K.L.B., Silva C.H.T.P., Silva J.O., Campos Rosa J.M., Santos C.B.R. (2018). An antioxidant potential, quantum-chemical and molecular docking study of the major chemical constituents present in the leaves of *Curatella americana* Linn. Pharmaceuticals.

[28] Cruz J.V., Serafim R.B., Silva G.M., Giuliatti S., Rosa J.M.C., Araújo Neto M.F., Leite F.H.A., Taft C.A., Silva C.H.T.P., Santos C.B.R. (2018). Computational design of new protein kinase 2 inhibitors for the treatment of inflammatory diseases using QSAR, pharmacophore-structure-based virtual screening and molecular dynamics. J. Mol. Model..

[29] Oxenoid K., Dong Y.S., Cao C., Cui T., Sancak Y., Markhard A.L., Grabarek Z., Kong L., Liu Z., Ouyang B. (2016). Architecture of the mitochondrial calcium uniporter. Nature.

[30] Dev J., Park D., Fu Q., Chen J., Ha H.J., Ghantous F., Herrmann T., Chang W., Liu Z., Frey G. (2016). Structural basis for membrane anchoring of HIV-1 envelope spike. Science.

[31] OuYang B., Xie S., Berardi M.J., Zhao X.M., Dev J., Yu W., Sun B., Chou J.J. (2013). Unusual architecture of the p7 channel from hepatitis C virus. Nature.

[32] Fu Q., Fu T.M., Cruz A.C., Sengupta P., Thomas S.K., Wang S., Siegel R.M., Wu H., Chou J.J. (2016). Structural basis and functional role of intramembrane trimerization of the Fas/CD95 death receptor. Mol. Cell.

[33] Li X.B., Wang S.Q., Xu W.R., Wang R.L. (2011). Novel Inhibitor Design for hemagglutinin against H1N1 influenza virus by core hopping method. PLoS ONE.

[34] Ma Y., Wang S.Q., Xu W.R., Wang R.L. (2012). Design novel dual agonists for treating type-2 diabetes by targeting peroxisome proliferator-activated receptors with core hopping approach. PLoS ONE.

[35] Chou K.C. (2004). Review: Structural bioinformatics and its impact to biomedical science. Curr. Med. Chem..

[36] Zimmerman H.J. (1999). Hepatotoxicity: The Adverse Effects of Drugs and Other Chemicals on the Liver.

[37] McMurtry R.J., Mitchell J.R. (1977). Renal and hepatic necrosis after metabolic activation of 2-substituted furans and thiophenes, including furosemide and cephaloridine. Toxicol. Appl. Pharmacol..

[38] Mitchell J.R., Potter W.Z., Hinson J.A., Jollow D.J. (1974). Hepatic necrosis caused by furosemide. Nature.

[39] Wiley R.A., Traiger G.J., Baraban S., Gammal L.M. (1984). Toxicity-distribution relationships among 3-alkylfurans in mouse liver and kidney. Toxicol. Appl. Pharmacol..

[40] Dalvie D.K., Kalgutkar A.S., Khojasteh-Bakht S.C., Obach R.S., O’Donnell J.P. (2002). Biotransformation reactions of five-membered aromatic heterocyclic rings. Chem. Res. Toxicol..

[41] Alvarez-Diez T.M., Zheng J. (2004). Mechanism-based inactivation of cytochrome P450 3A4 by 4-ipomeanol. Chem. Res. Toxicol..

[42] Gordon W.P., Forte A.J., McMurtry R.J., Gal J., Nelson S.D. (1982). Hepatotoxicity and pulmonary toxicity of pennyroyal oil and its constituent terpenes in the mouse. Toxicol. Appl. Pharmacol..

[43] Peterson L.A., Naruko K.C., Predecki D.P. (2000). A reactive metabolite of furan, *cis*-2-butene-1,4-dial, is mutagenic in the Ames assay. Chem. Res. Toxicol..

[44] Hamadeh H.K., Jayadev S., Gaillard E.T., Huang Q., Stoll R., Blanchard K., Chou J., Tucker C.J., Collins J., Maronpot R. (2004). Integration of clinical and gene expression endpoints to explore furan-mediated hepatotoxicity. Mutat. Res..

[45] Nelson S.D. (2001). Structure toxicity relationships—How useful are they in predicting toxicities of new drugs?. Adv. Exp. Med. Biol..

[46] Benjamin S.B., Ishak K.G., Zimmerman H.J., Grushka A. (1981). Phenylbutazone liver injury: A clinical-pathologic survey of 23 cases and review of the literature. Hepatology.

[47] Gaisford W. (1962). Fatality after oxyphenbutazone in Still’s disease. Br. Med. J..

[48] Kunze K.D., Porst H., Tschopel L. (1985). Morphology and pathogenesis of liver injury produced by dihydralazine, propranolol and ketophenylbutazone. Zentralbl. Allg. Pathol. Pathol..

[49] Wiggins J., Scott D.L. (1981). Hepatic injury following feprazone therapy. Rheumatol. Rehabil..

[50] Muller H.G. (1981). Jaundice following sulfinpyrazone administration. Dtsch. Med. Wochenschr..

[51] Kari F., Bucher J., Haseman J., Eustis S., Huff J. (1995). Long-term exposure to the anti-inflammatory agent phenylbutazone induces kidney tumors in rats and liver tumors in mice. Jpn. J. Cancer Res..

[52] Kayser D., Schlede E. (2001). Chemikalien und Kontaktallergie—Eine bewertende Zusammenstellung.

[53] Wahlberg J.E., Boman A. (1985). Guinea pig maximization test. Curr. Problems Dermatol..

[54] Rycroft R.J.G., Wilkinson J.D., Champion R.H., Burton J.L., Ebling F.J.G. (1991). Irritants and sensitisers. Textbook of Dermatology.

[55] Parr R.G., Yang W. (1989). Density Functional Theory of Atoms and Molecules.

[56] Frisch M.J., Trucks G.W., Schlegel H.B., Scuseria G.E., Robb M.A., Cheeseman J.R., Scalmani G., Barone V., Mennucci B., Petersson G.A. (2009). Gaussian 09 Revision A.02.

[57] Horton W., Peerannawar S., Török B., Török M. (2018). Theoretical and experimental analysis of the antioxidant features of substituted phenol and aniline model compounds. Struct. Chem..

[58] Becke A.D.J. (1993). Density-funtional thermochemistry. III. The role of exact exchange. Chem. Phys..

[59] Lee C., Yang W., Parr R.G. (1998). Development of the Colle-Salvetti correlation-energy formula into a functional of the electron density. Phys. Rev..

[60] Santos C.B.R., Lobato C.C., Braga F.S., Costa J.S., Favacho H.A.S., Carvalho J.C.T., Macedo W.J.C., Brasil D.S.B., Silva C.H.T.P., Hage-Melim L.I.S. (2015). Rational design of antimalarial drugs using molecular modeling and statistical analysis. Curr. Pharm. Desig..

[61] (2015). Discovery Studio Modeling Environment.

[62] Macêdo W.J.C., Braga F.S., Santos C.F., Costa J.S., Melo G.S., Mello M.N., Sousa D.S., Carvalho J.C.T., Brasil D.S.B., Santos C.B.R. (2015). Antimalarial artemisinins derivatives study: Molecular modeling and multivariate analysis: PCA. HCA. KNN. SIMCA and SDA. J. Comp. Threoret. Nanosc..

[63] Federico L., Santos C.B.R., Lobato C., Gomes J., Rosa J.M.C., Silva C.H.T.P. (2017). Ligand- and structure-based drug design of novel calcium channel blockers. J. Comp. Threoret. Nanosc..

[64] Padilha E.C., Serafim R.B., Sarmiento D.Y.R., Santos C.F., Santos C.B.R., Silva C.H.T.P. (2016). New PPARα/γ/δ optimal activator rationally designed by computational methods. Braz. Chem. Soc..

[65] Pereira A.L.E., Santos G.B., Franco M.S.F., Federico L.B., Silva C.H.T.P., Santos C.B.R. (2018). Molecular modeling and statistical analysis in the design of derivatives of human dipeptidyl peptidase IV. J. Biomol. Struct. Dyn..

[66] Costa J.S., Ramos R.S., Costa K.S.L., Brasil D.S.B., Silva C.H.T.P., Ferreira E.F.B., Borges R.S., Campos J.M., Macêdo W.J.C., Santos C.B.R. (2018). An in silico study of the antioxidant ability for two caffeine analogs using molecular docking and quantum chemical methods. Molecules.

[67] (2007). Derek for Windows.

[68] Rindings J.E., Barratt M.D., Cary R. (1996). Computer prediction of possible toxic action from chemical structure, an update of the DEREK system. Toxicology.

[69] Barcellos M.P., Santos C.B.R., Federico L.B., Almeida P.F., Silva C.H.T.P., Taft C.A. (2018). Pharmacophore and structure-based drug design, molecular dynamics and admet/tox studies to design novel potential pad4 inhibitors. J. Biomol. Struct. Dyn..

