# Supplementary material for: Toward of Safer Phenylbutazone Derivatives by Exploration of Toxicity Mechanism
Source: Molecules. 2019 Jan 1;24(1):143. doi: 10.3390/molecules24010143 (PMC6337259; doi:10.3390/molecules24010143)
Supplement: Supplementary file 1 [file molecules-24-00143-s001.pdf]

molecules

MDPI

Article

Toward of safer phenylbutazone derivatives by exploration of toxicity mechanism

Rosivaldo S. Borges [1,2,*], Ivanete C. Palheta [1], Sirlene S. B. Ota [1,2], Roberto B. Morais [1,2], Valéria A. Barros [1,2], Ryan S. Ramos [3], Rai C. Silva [2,4], Josivan da S. Costa [4], Carlos H. T. P. Silva [5] Joaquín M. Campos [6] and Cleydson B. R. Santos [2,3,4,*]

1 Núcleo de Estudos e Seleção de Moléculas Bioativas – NESBio, College of Pharmacy, Health Sciences Institute, Federal University of Pará, Belém 66075-110, PA, Brazil; lqfmed@gmail.comivapalheta@gmail.com (I.C.P.); sayuriota@gmail.com (S.S.B.O.); robertomorais16@hotmail.com.br (R.B.M.); valbarros43@yahoo.com.br (V.A.B.)

2 Programa de Pós-Graduação em Química Medicinal e Modelagem Molecular, Health Science Institute, Federal University of Pará, Belém 66075-110, PA, Brazil; camposchemistry@gmail.com (R.C.S.)

3 Programa de Pós-Graduação em Biodiversidade e Biotecnologia – Rede BIONORTE, Federal University of Amapá, Macapá 68902-280, AP, Brazil; ryanquimico@hotmail.com (R.S.R.)

4 Laboratorio de Modelagem e Química Computacional – LMQC, Federal University of Amapá, Department of Biological Sciences. Rod. Juscelino Kubitschek, Km 02, Macapá 68902-280, AP, Brazil; josivan.chemistry@gmail.com (J.d.S.C.)

5 Laboratório Computacional de Química Farmacêutica, Faculdade de Ciências Farmacêuticas de Ribeirão Preto, University of Sao Paulo, São Paulo 14040-903, SP, Brazil; tomich@fcfrp.usp.br (C.H.T.P.S.)

6 Department of Pharmaceutical Organic Chemistry, University of Granada, 18071 Granada, Spain; jmcampos@ugr.es (J.M.C.)

* Correspondence: lqfmed@gmail.com (R.S.B.); breno@unifap.br (C.B.R.S.); Tel.: +55-96-4009-2699 (C.B.R.S.)

SUPPLEMENTARY MATERIAL

Table S1. Interactions between ligands with therapeutic targets COX-2 (organism *Mus musculus*).

Table S2. Interactions between indometacin and refocoxib ligands with therapeutic targets COX-2 (organism *Mus musculus*).

Table S3. Interactions between ligands with therapeutic targets COX-2 (organism *Homo sapiens*)

Table S4. Interactions between Indometacin and Refocoxib ligands with therapeutic targets COX-2 (organism *Homo sapiens*)

Table S1. Interactions between ligands with therapeutic targets COX-2 (organism *Mus musculus*).

Compounds	Aminoacid	Distance (Å)	Type	Binding Free Energy (kcal/mol)
01 vs. 4COX	Arg120	3.51002	Pi-Cation	−8.79
	Val349	5.11613	Pi-Alkyl	
	Leu352	4.46599	Pi-Alkyl	
	Phe381	5.43650	Pi-Alkyl	
	Tyr385	3.93660	Pi-Sigma	
	Trp387	4.62120	Pi-Alkyl	
	Val523	3.84629	Pi-Sigma	
	Ala527	4.13700	Pi-Alkyl	
	Ala527	2.89407	Carbon Hydrogen Bond	
	Ser530	3.20143	Carbon Hydrogen Bond	
	Leu531	5.37547	Pi-Alkyl	
13 vs. 4COX	Val349	4.84218	Alkyl	−11.05
	Ser353	3.60900	Pi-Sigma	
	Tyr355	4.29553	Pi-Alkyl	
	Leu359	4.96936	Alkyl	
	Leu384	4.20658	Alkyl	
	Tyr385	3.79286	Pi-Alkyl	
	Met522	5.05536	Pi-Sulfur	
	Met522	4.89886	Amide-Pi Stacked	
	Val523	3.11434	Pi-Sigma	
14 vs. 4COX	Val349	3.80477	Alkyl	−11.23
	Ser353	3.63124	Carbon Hydrogen Bond	
	Phe381	4.91784	Pi-Alkyl	
	Leu384	4.84624	Alkyl	
	Tyr385	3.63736	Pi-Alkyl	
	Met522	5.18737	Alkyl	
	Val523	3.46937	Pi-Sigma	
	Ala527	4.686149	Amide-Pi Stacked	
15 vs. 4COX	Val349	5.45496	Alkyl	−10.01
	Ser353	3.51557	Pi-Sigma	
	Tyr385	4.95721	Pi-Pi T-shaped	
	Met522	4.80672	Pi-Sulfur	
	Val523	3.15646	Pi-Sigma	
	Gly526	4.68615	Amide-Pi Stacked	
	Ala527	4.68615	Amide-Pi Stacked	

Table S2. Interactions between Indometacin and Refocoxib ligands with therapeutic targets COX-2 (organism *Mus musculus*).

Compounds	Aminoacid	Distance (Å)	Type	Binding Free Energy (kcal/mol)
Indometacin vs. 4COX	Val349	4.17336	Alkyl	−10.70
	Val349	3.95422	Pi-Sigma	
	Leu352	5.13778	Pi-Alkyl	
	Leu352	2.84374	Carbon Hydrogen Bond	
	Ser353	2.91033	Carbon Hydrogen Bond	
	Met522	4.22540	Alkyl	
	Val523	4.18228	Pi-Alkyl	
	Val523	5.48361	Pi-Alkyl	
	Ala527	5.23742	Pi-Alkyl	
	Ala527	3.92867	Pi-Sigma	
	Ala527	3.82751	Alkyl	
	Ser530	3.1636	Hydrogen Bond	
	Leu531	4.01432	Alkyl	
Refocoxib vs. 4COX	Val349	3.77795	Pi-Sigma	−9.65
	Leu352	4.89044	Pi-Alkyl	
	Tyr385	3.61598	Pi-Sigma	
	Val523	3.52599	Pi-Sigma	
	Gly526	3.57032	Amide-Pi Stacked	
	Ala527	4.68927	Pi-Alkyl	
	Ala527	3.59451	Pi-Sigma	
	Leu531	5.4851	Pi-Alkyl	

Table S3. Interactions between ligands with therapeutic targets COX-2 (organism *Homo sapiens*)

Compounds	Aminoacid	Distance (Å)	Type	Binding Free Energy (kcal/mol)
01 vs. 5KIR	His90	4.02775	Pi-Alkyl	−8.40
	Val349	4.2394	Pi-Alkyl	
	Leu352	3.59932	Pi-Sigma	
	Val523	4.62394	Alkyl	
	Ala527	3.87217	Pi-Sigma	
	Leu531	4.93981	Pi-Alkyl	
07 vs. 5KIR	Val116	4.70465	Alkyl	−9.75
	Arg120	3.00485	Conventional Hydrogen Bond	
	Tyr355	2.19613	Conventional Hydrogen Bond	
	Leu384	4.45801	Alkyl	
	Ala516	3.98735	Alkyl	
	Met522	4.33431	Alkyl	
	Val523	3.26309	Pi-Sigma	
08 vs. 5KIR	His90	5.32308	Pi-Alkyl	−8.25
	Leu352	2.97837	Pi-Lone Pair	
	Tyr355	4.03608	Pi-Alkyl	
	Val523	3.92022	Alkyl	
	Ala527	3.64342	Alkyl	
	Leu531	4.43391	Alkyl	
14 vs. 5KIR	Tyr355	5.29323	Pi-Alkyl	−9.91
	Phe381	4.76475	Pi-Alkyl	
	Leu384	4.7156	Alkyl	
	Tyr385	3.94543	Alkyl	
	Trp387	5.25307	Pi-Alkyl	
	Ala516	4.6385	Pi-Alkyl	
	Val523	4.72767	Pi-Alkyl	
	Ala527	3.59612	Alkyl	
15 vs. 5KIR	Val349	5.02376	Alkyl	−9.56
	Val523	3.51509	Pi-Sigma	
	Gln192	2.76587	Halogen (Cl. Br. I)	
	Ser353	3.61607	Pi-Sigma	
	Tyr355	2.30645	Conventional Hydrogen Bond	
	Ala516	3.51741	Alkyl	
	Val523	3.33452	Pi-Sigma	
	Gly526	4.15184	Amide-Pi Stacked	
	Ala527	3.76613	Pi-Sigma	
	Ser530	2.66011	Conventional Hydrogen Bond	

Table S4. Interactions between Indometacin and Refocoxib ligands with therapeutic targets COX-2 (organism *Homo sapiens*)

Compounds	Aminoacid	Distance (Å)	Type	Binding Free Energy (kcal/mol)
Refocoxibe vs. 5KIR	His90	2.64213	Conventional Hydrogen Bond	−10.14
	Val349	4.41806	Pi-Alkyl	
	Leu352	5.44011	Pi-Alkyl	
	Arg513	2.55259	Carbon Hydrogen Bond	
	Arg513	3.07173	Carbon Hydrogen Bond	
	Arg513	2.37819	Conventional Hydrogen Bond	
	Phe518	5.82249	Pi-Pi Stacked	
	Val523	3.80966	Pi-Alkyl	
	Ala527	4.95368	Pi-Alkyl	
	Ala527	3.97589	Pi-Alkyl	
	Ala527	2.61541	Carbon Hydrogen Bond	
	Ser530	2.84906	Carbon Hydrogen Bond	
Indometacin vs. 5KIR	Arg120	2.89914	Conventional Hydrogen Bond	−8.11
	Gln192	2.76587	Halogen (Cl. Br. I)	
	Val349	5.00586	Pi-Alkyl	
	Val349	5.39105	Alkyl	
	Ser353	3.61607	Pi-Sigma	
	Tyr355	4.16467	Pi-Alkyl	
	Tyr355	2.30645	Conventional Hydrogen Bond	
	Ala516	3.51741	Alkyl	
	Ile517	5.32659	Alkyl	
	Val523	3.33452	Pi-Sigma	
	Val523	4.98848	Pi-Alkyl	
	Val523	4.75967	Pi-Alkyl	
	Gly526	4.15184	Amide-Pi Stacked	
	Ala527	3.92327	Pi-Sigma	
	Ala527	3.76613	Pi-Sigma	
	Ser530	2.66011	Conventional Hydrogen Bond	
